# Oral manifestations of pediatric cancer patients receiving chemotherapy and dental awareness among their parents-a cross-sectional study

**DOI:** 10.1186/s12903-026-07983-7

**Published:** 2026-03-27

**Authors:** Amira Mohammed Al-Sayed, Sherif Bahgat El-Tawil, Youssef Madney, Yasmin Mohamed Yousry

**Affiliations:** 1 Faculty of Dentistry, Cairo University, Giza, Egypt; 2 Pediatric Oncology, National Cancer Institute, Cairo University, Giza, Egypt

**Keywords:** Awareness, Chemotherapy, Oral Health, Pediatric Cancer, Oral Manifestations

## Abstract

**Background:**

Oral cavity lesions are among the most frequent complications of antineoplastic chemotherapy. This study investigated oral manifestations in children undergoing chemotherapy and assessed dental awareness among their parents.

**Materials and methods:**

The study sample included 441 pediatric cancer patients aged 1- to 14-years-old who were receiving chemotherapy. Oral assessments were systematically conducted on eligible patients using the Oral Assessment Guide (OAG) criteria and the World Health Organization Mucositis (WHO) grading system to ensure objective measurement. A detailed survey evaluating parental awareness of dental care practices was distributed and completed by caregivers accompanying the children. Subsequently, all collected data and values underwent a comprehensive statistical analysis.

**Results:**

Regarding the OAG score, oral manifestations were observed in children who underwent chemotherapy treatment with a mean score of 11.66. Statistical analysis revealed no significant correlation between age or gender in relation to oral manifestations. A significant association was found between the type of cancer and saliva score (*p*-value 0.005). Most parents reported that their children experienced oral complications, received nutritional advice from a pediatrician or dietitian, and showed an interest in their child’s dental care. Regarding the relationship between oral manifestation scores and frequency of teeth brushing per day, children who brushed their teeth only once had significantly lower scores in voice (p-value 0.018) and saliva (p-value 0.026) compared to other children.

**Conclusions:**

Children undergoing chemotherapy frequently experience oral complications, with tooth debris and dry lips being the most commonly observed manifestations. Although parents demonstrated an interest in their children’s dental care, they lacked sufficient awareness of appropriate oral health practices.

**Trial registration:**

Clinicaltrials.gov registration number was: NCT05224882. Clinicaltrials.gov registration date was: November 26, 2022.

## Background

Cancer is a complex pathological condition characterized by multiple alterations in cellular physiology that ultimately lead to malignant tumors. The biological hallmark of the disease is abnormal cellular proliferation (neoplasia) [1].

Childhood cancer, defined as cancer in individuals aged 0–14 years, represents a small proportion of the global cancer burden. However, 84% of childhood malignancies occur in low- and middle-income countries (LMICs), where approximately 90% of the world’s children reside, and access to healthcare is often limited [2].

Cancer is a potentially life-threatening disease that has been considered incurable in many cultures. The term “cancer” has negative connotations such as fear, agony, loss of hope, and suffering. Receiving such a diagnosis may be an incredibly stressful event that affects both the patients and their families physically as well as emotionally [3]. Childhood cancer is a significant burden as well as an awful experience for any family, and knowing that there is hope makes the struggle bearable [4].

According to the Global Cancer Observatory (2020), Egypt is a lower-middle-income country with the second-highest estimated number of incident childhood cancer cases in the WHO Eastern Mediterranean Region. Over the past century, treatment outcomes for pediatric tumors have improved dramatically, and treatment paradigms have shifted from surgery alone to multimodal methods that include chemotherapy and radiation [5].

Recent advancements in cancer treatment, such as chemotherapy, have significantly improved survival outcomes among children and adults. However, patients frequently must deal with the significant, unfavorable side effects of their life-saving treatment during treatment and later in life. Chemotherapeutic agents typically target and damage DNA, resulting in mutations or genomic instability, a significant feature of both cancer and aging [6].

Despite significant advances in chemotherapy, oral problems remain common. Chemotherapy-induced somatotoxicities are a frequent side effect associated with pediatric cancer treatment. Oral complications of chemotherapy include oral mucositis, gingival bleeding, xerostomia, viral and fungal infections, with these manifestations occurring more frequently among children compared to adult populations. These complications cause significant discomfort, nutritional challenges, severe malnutrition, prolonged hospitalization, and potential bloodstream infection, resulting in a substantial decline in a patient’s quality of life and overall treatment compliance [7].

The majority of hospitalized children receiving chemotherapy exhibited poor oral health conditions; therefore, preventing oral complications in children and adolescents receiving oncological therapy remains essential, since oral lesions caused by this treatment significantly worsen their overall medical condition and elevate serious infection susceptibility, additionally complicating required dental interventions when clinically necessary [8].

Parents of children receiving cancer treatment, particularly chemotherapy, who are in an acute immunosuppressive phase, commonly neglect dental care. As a result, the importance of consistently sending pediatric cancer patients to hospital-based pediatric dental clinics for urgent dental care must be emphasized [9].

Preventing complications is far more crucial than treating them. All patients initiating systemic anticancer treatment should undergo a comprehensive oral examination and receive appropriate prophylaxis [10]. Therefore, the principal objective of this research was to report the oral manifestations of pediatric cancer patients undergoing chemotherapy and to assess their parents’ dental awareness, so that the proper interventions can be implemented to prevent more serious complications. To our knowledge, this study is unique in integrating the perspectives of children, their parents or caregivers, and medical professionals, providing a comprehensive overview of oral complications in pediatric cancer patients undergoing chemotherapy. It emphasizes not only the objective clinical findings but also the subjective parental perceptions, aspects that are rarely assessed together.

## Materials and methods

### Study design and sample characteristics

The study was an observational descriptive cross-sectional study. The study was conducted at the outpatient and inpatient clinics of the Pediatric Oncology Department at the National Cancer Institute in Cairo, Egypt, among patients currently receiving chemotherapy and their parents. The study was conducted between August 2023 and July 2024.

The trial was registered on www.clinicaltrials.gov with the protocol ID NCT05224882. The Ethical Committee, Faculty of Dentistry, Cairo University (REC), approved this study with approval number 5-7-22, and the Institutional Review Board (IRB) at the National Cancer Institute, Cairo University, Egypt, with approval number 2301-407-0013.

The purpose and procedures of the study were explained simply to the legal guardians, and written consent was obtained from them after the inclusion criteria were met. Additionally, children capable of comprehension received an age-appropriate explanation of the procedures, and verbal assent was obtained prior to examination.

### Eligibility criteria

#### Inclusion criteria

- Children between the ages of 1 and 14years.
- Children were receiving chemotherapy for hematological cancer or solid tumors.
- Caregivers who could understand the aim of the study and were willing to participate in the study.

#### Exclusion criteria

- Children previously received radiotherapy to the head and neck region.
- Children were suffering from oral cancer.
- Children presented with other oral disorders such as leukoplakia.

### Sample size

To conduct a statistical test of the research question regarding the oral manifestations observed in pediatric cancer patients undergoing chemotherapy, a power analysis was designed to ensure adequate power to apply a statistical test. Using a 95% confidence interval, a 3% margin of error, and a finite population correction, the predicted sample size (n) was 441 cases, based on the findings of a prior study by Hendrawati et al. [11], which reported a prevalence of mucositis of 88.3%. Epi Info for Windows 7.2 was used to calculate the sample size.

### Data collection method

#### Primary outcome data

Clinical examinations were carried out in the Pediatrics Department at the National Cancer Institute, in the chemotherapy room, and in the inpatient clinics. The study examined 441 pediatric patients receiving chemotherapy. Due to the inability to conduct the evaluations under ideal lighting conditions in the dental office, the patients were assessed in their beds under adapted lighting conditions, using flashlights.

#### Patient assessment chart

The principal investigator was responsible for recording the data on a specially designed patient assessment chart on the day of examination. The recording chart was composed of three sections.

The first section included the personal history, which consisted of demographic data, past and present medical history (including cancer diagnoses, illness duration, hospitalizations, and any surgeries performed), as well as past dental history.

The second section included the extra-oral examination of the patient and the intra-oral examination using the Oral Assessment Guide and the World Health Organization Mucositis Scale [12], during which the patient was evaluated for any abnormal changes. All participants were examined using latex gloves, masks, plain mouth mirrors, sterile gauze, and wooden tongue depressors. The score for each scale was chosen by the principal investigator and recorded in the patient assessment chart.

The third section included the habits performed by the patient, which consisted of oral hygiene habits and fluoride application.

Scales used for assessment of oral conditions:

### A. Oral Assessment Guide (OAG)

This instrument, adapted from Eilers et al. [13], allows for the evaluation of eight items: swallowing, lips, tongue, saliva, mucous membrane, gingivae, teeth, and voice, based on the oral health compromise scales. Each anatomical site is assigned values ranging from 1 to 3 for each item [12]:

1.  For conditions in which normality is confirmed.
2. For the confirmation of mild-to-moderate changes in epithelial integrity or function.
3.  For a severe compromise.

**Fig. 1:**
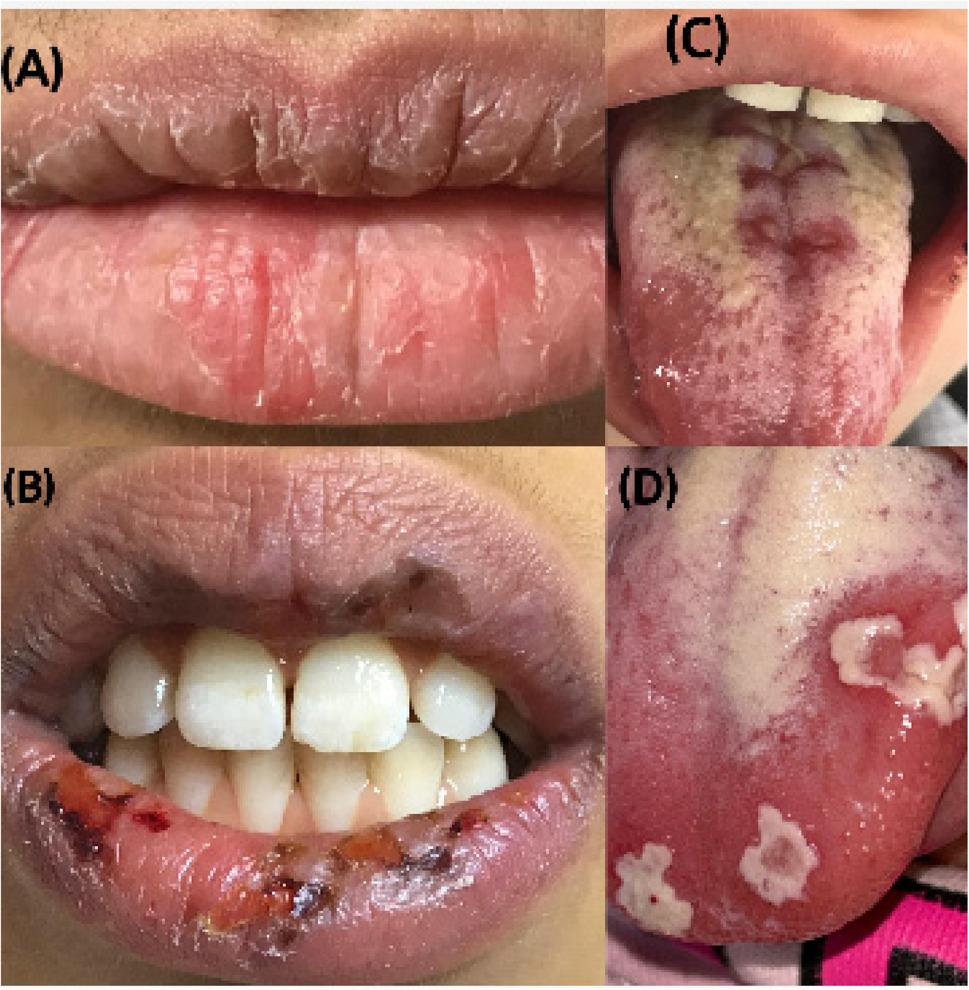
Clinical pictures for patients with oral manifestations involving the lip and tongue. **A** Dry and cracked lip score (2). **B** Bleeding lips score (3). **C** Cracked tongue Score (3). **D **Coated tongue with redness Score (2)

**Fig. 2:**
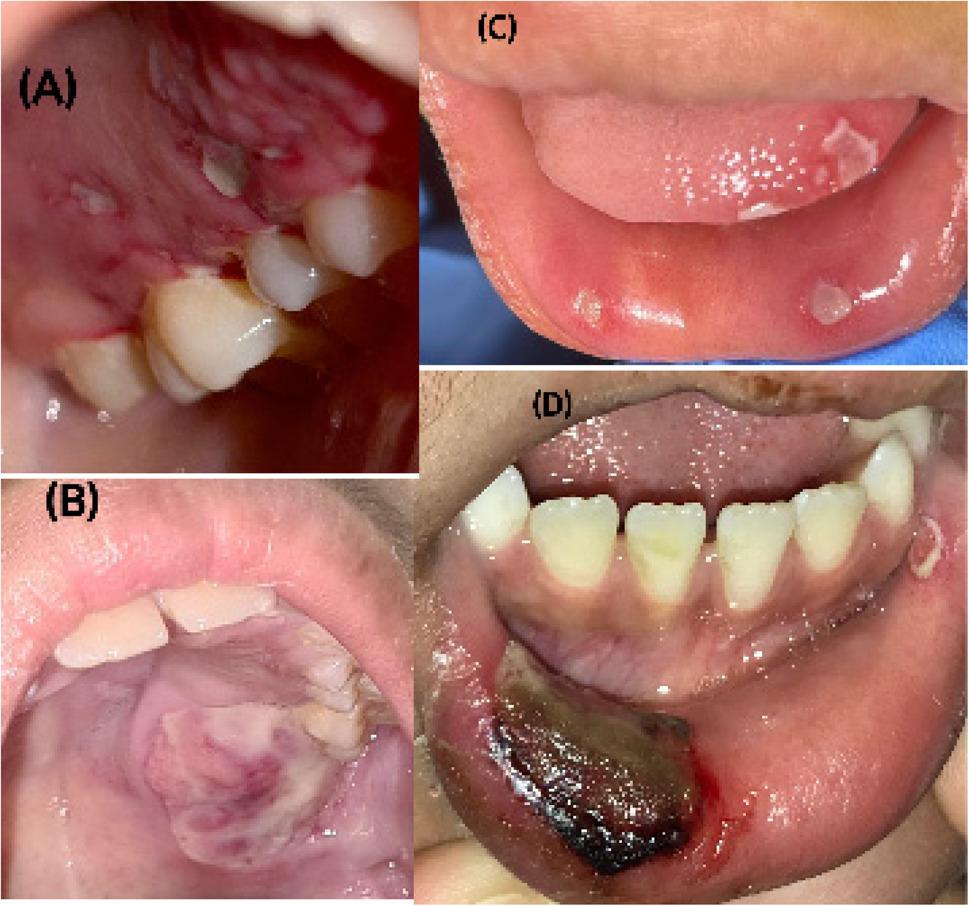
Clinical pictures for patients with oral manifestations involving the mucosa. **A** Reddened and coated palatal mucous membrane without ulceration. **B** Ulcerated palatal mucous membrane. **C** Ulcerated labial mucous membrane without bleeding. **D** Ulcerated labial mucous membrane with bleeding

**Fig. 3:**
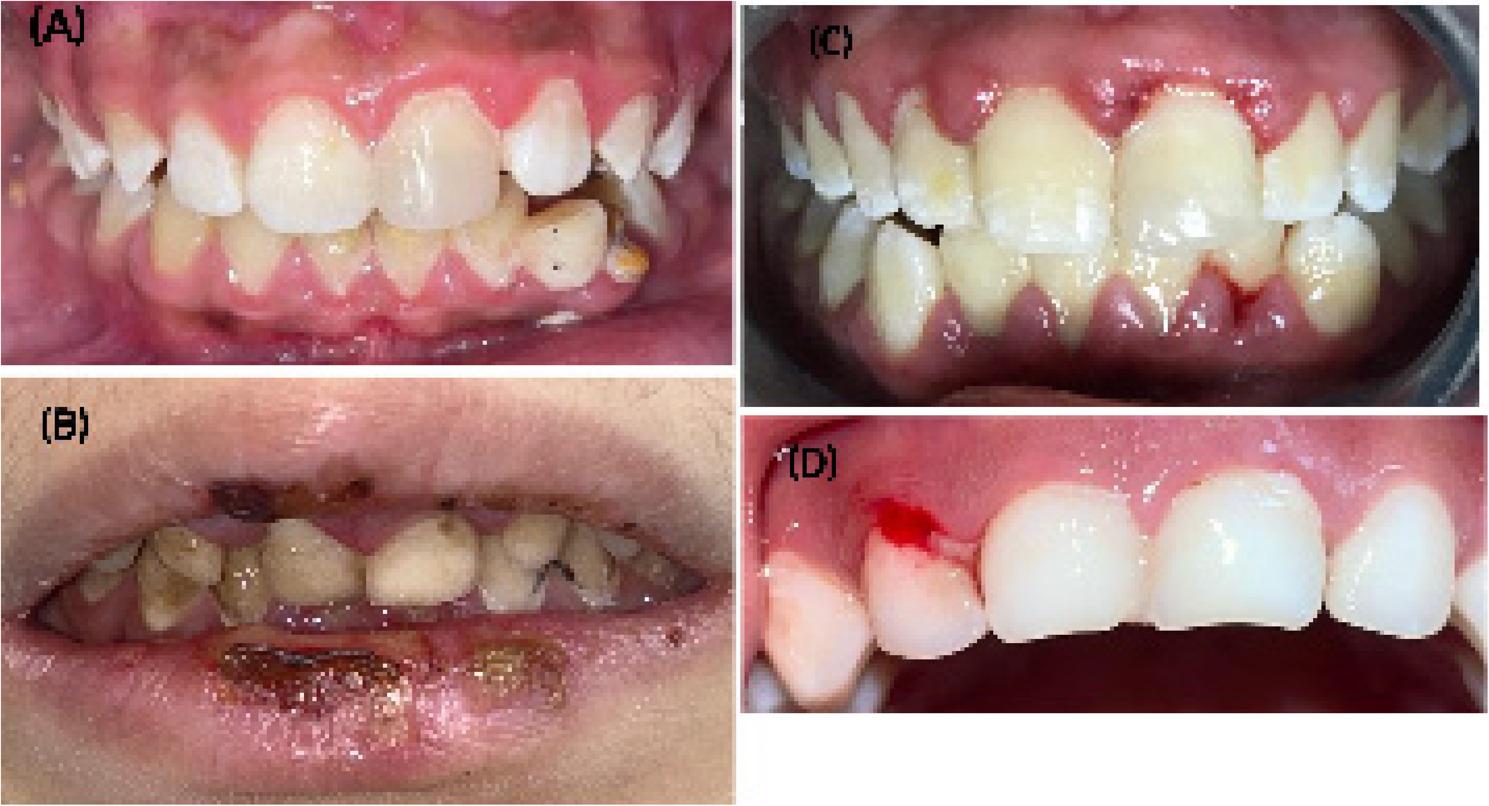
Clinical pictures for patients with oral manifestations involving teeth and gingiva. **A** Plaque or debris in localized areas. **B** Plaque or debris along the gum line. **C** Edematous gingiva with redness. **D** Spontaneous bleeding

Finally, the total verified mucositis ranges from 8 to 24; there is no cutting point between these values for estimating mucositis [14]. Therefore, this study was accompanied by the WHO mucositis scale for assessing the mucositis score if present. Figs. 1, 2 and 3 showed clinical pictures for some cases scored using the OAG scale.

### B. World Health Organization Mucositis Scale (WHO)

The WHO rating scale assesses oral mucositis based on anatomical, symptomatic, and functional factors (12). OM is graded as:

(0) Normal.
(1) Mild focal change, which appears as erythematous areas that are not yet painful or sensitive, and oral intake is tolerated.
(2) Painless ulcers, erythema, and only mild pain.
(3) Painful erythema, edema, or ulcers of depth > 2 mm covering less than half of the mucosa; no bleeding; and oral intake of only a liquid diet is possible.
(4) Severe pain; bleeding; and an inability to swallow nutrition, which necessitates parenteral and enteral nutrition support.

**Fig. 4:**
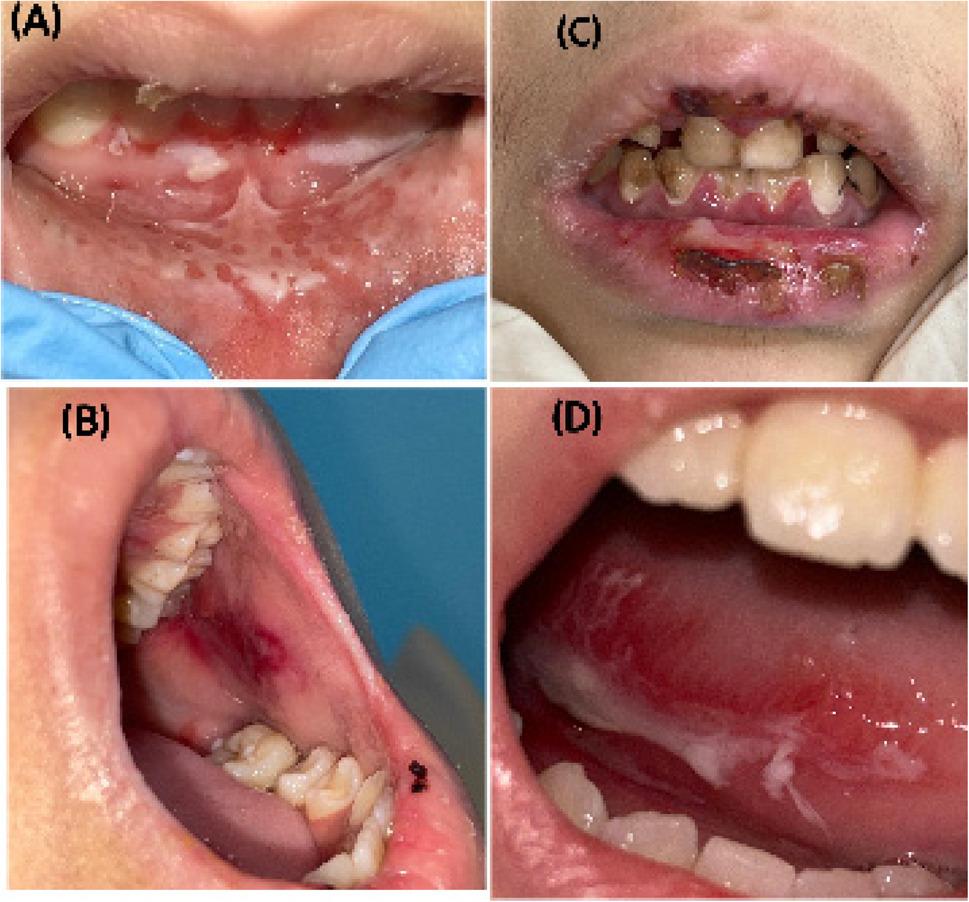
Clinical pictures for patients with mucositis. **A** Mucositis grade I: mild oral soreness and erythema. **B** Mucositis grade II: moderate oral erythema, solid diet tolerated. **C** Mucositis grade III: severe oral ulcers; only a liquid diet is tolerated. **D** Mucositis grade IV oral alimentation is impossible

Figure 4 shows clinical pictures for some cases scored by the WHO scale with different grades of mucositis.

### Secondary outcome data

#### Parental questionnaire

A questionnaire assessing awareness towards dental care, consisting of 15 closed-ended questions, was distributed and completed by 441 parents of pediatric patients receiving chemotherapy, validated by Gupta et al. [15]. The questionnaire assessed oral hygiene practices, including toothbrushing frequency, type of toothpaste (brand and whether it was fluoridated/non-fluoridated), and the use of any other oral hygiene measures. The questionnaire also assessed dietary habits, including the type of diet and consumption of sweetened beverages/ snacks. In addition to the parental attitudes, the frequency of dental visits and any previous prophylactic dental treatment were assessed. After completing the questionnaire, parents were provided with instructions on oral hygiene procedures, diet, and fluoride application that should be followed during chemotherapy.

#### Addressing a potential source of bias

- Selection Bias: was avoided by including all the patients fulfilling the inclusion criteria on the day of visit.
- Information Bias: was avoided by using a validated scale for clinical assessment, as well as a validated dental awareness questionnaire for the parents, without guiding them to specific answers.
- Blinding Bias: The data was sent to the statistician, labelled to avoid any bias.
- Reporting Bias: was avoided by reporting all the data assessed.

#### Statistical analysis

Frequency and percentage values were used to display both ordinal and categorical data. The mean, standard deviation (SD), median, and interquartile range (IQR) values were used to display the numerical data. By reviewing the data distribution and applying the Shapiro-Wilk test, the normality of the data was examined. While other numerical data were non-parametric, age data had a normal distribution. Kruskal-Wallis’s test, followed by Dunn’s post hoc test, was used to examine numerical and ordinal data for various associations. Spearman’s rank-order correlation coefficient was used for correlation analysis. For all tests, the significance level was set at *p* < 0.05. R statistical analysis software, version 4.3.2 for Windows, was used to conduct the statistical analysis.

## Results

### Demographic data

The study was conducted on a total of 441 children (255 males and 186 females) with a mean age of (7.64 ± 3.46) years, the majority of whom were predominantly affected with Acute Lymphoblastic Leukemia (hematological lesions) and Osteosarcoma (solid lesions), as shown in Table 1.

**Table 1:** Demographic data

Parameter			Value
Gender [n (%)]		Male	255 (57.82%)
		Female	186 (42.18%)
Age (years)		Mean ± SD	7.64 ± 3.46
		Median (IQR)	7.00 (5.00)
Cancer diagnosis [n (%)]	Hematological	Acute Lymphoblastic Leukemia	186 (64.58%)
		Non-Hodgkin’s Lymphoma	8 (2.78%)
		Atypical Teratoid Rhabdoid	4 (1.39%)
		Burkitt’s Lymphoma	33 (11.46%)
		Hodgkin’s Lymphoma	7 (2.43%)
		Acute Myeloid Leukemia	43 (14.93%)
		B-Cell Lymphoma	7 (2.43%)
	Solid	Osteosarcoma	47 (30.72%)
		Medulloblastoma	14 (9.15%)
		Primitive Neuro-ectodermal tumor	2 (1.31%)
		Neuroblastoma	40 (26.14%)
		Ewing’s Sarcoma	9 (5.88%)
		Wilm’s tumor	17 (11.11%)
		Gastric Cancer	6 (3.92%)
		Neuroendocrine tumor	2 (1.31%)
		Infantile Fibrosarcoma	3 (1.96%)
		Germ Cell Tumor	4 (2.61%)
		Rhabdomyosarcoma	6 (3.92%)
		Ovarian Cancer	1 (0.65%)
		Optic Nerve Glioma	1 (0.65%)
		Hepatoblastoma	1 (0.65%)

### Oral manifestations

Regarding the Voice, Swallow, Lips, Tongue, Saliva, Mucous Membrane, and Gingiva scores, the majority scored (1), while regarding teeth, the majority scored (2). Regarding the Mucositis score, the majority (57.60%) took score (0), (17.46%) took score (1), (10.66%) took score (2), (9.30%) took score (3), and (4.99%) took score (4), as shown in Tables 2 and 3.

**Table 2:** Oral assessment guide scores

Parameter	*n* (%)					
	Score (1)	Score (2)			Score (3)	
Voice	377 (85.49%)	41 (9.30%)			23 (5.22%)	
Swallow	296 (67.12%)	100 (22.68%)			45 (10.20%)	
Lips	196 (44.44%)	172 (39.00%)			73 (16.55%)	
Tongue	290 (65.76%)	126 (28.57%)			25 (5.67%)	
Saliva	337 (76.42%)	74 (16.78%)			30 (6.80%)	
Mucous Membrane	299 (67.80%)	125 (28.34%)			17 (3.85%)	
Gingiva	257 (58.28%)	166 (37.64%)			18 (4.08%)	
Teeth	156 (35.37%)	224 (50.79%)			61 (13.83%)	

Parameter	Mean	95% CI		SD	Min	Max
		Lower	Upper			
Total OAG score	11.66	11.32	11.99	3.62	8.00	24.00

**Table 3:** Mucositis score

Parameter	*n* (%)				
	Score (0)	Score (1)	Score (2)	Score (3)	Score (4)
Mucositis	254 (57.60%)	77 (17.46%)	47 (10.66%)	41 (9.30%)	22 (4.99%)

### Parental dental awareness

For questions (1), (2), (3), (4), (5), (6), (8), (10), (12), and (13), many respondents chose “|No” while for other questions they chose “Yes”. For question (15) regarding the frequency of teeth brushing per day, most of the participants chose “Never” as shown in Table 4.

**Table 4:** Answers to parental dental awareness questionnaire (Q1:14)

Question		*n* (%)	
		No	Yes
1- Whether your child’s physician recommends not brushing your child’s teeth during chemotherapy periods?		368 (83.45%)	73 (16.55%)
2- Whether taken your child to a dentist before starting chemotherapy?		419 (95.01%)	22 (4.99%)
3- History of any fluoride application?		434 (98.41%)	7 (1.59%)
4- Has your child ever complained of tooth pain during chemotherapy periods?		246 (55.78%)	195 (44.22%)
5- Whether referred your child to a pediatric dentist to treat emergency dental pain or abscess?		377 (85.49%)	64 (14.51%)
6- History of bleeding in child’s oral cavity?		345 (78.23%)	96 (21.77%)
7- Whether complaints of mouth sores during chemotherapy periods?		107 (24.26%)	334 (75.74%)
8- Whether consumes sweetened beverages or eating snacks between meals?		232 (52.61%)	209 (47.39%)
9- Whether given dietary counseling by the pediatrician or dietitian?		108 (24.49%)	333 (75.51%)
10- Have you received any preventive dental advice during therapy?		293 (66.44%)	148 (33.56%)
11- Whether your child complains of dry mouth during therapy?		199 (45.12%)	242 (54.88%)
12- Whether using mouthwash for your child?		296 (67.12%)	145 (32.88%)
13- If yes, does your child use the mouthwash daily?		351 (79.59%)	90 (20.41%)
14- Are you interested in dental care for your child?		108 (24.49%)	333 (75.51%)

Question	*n* (%)		
	Never	Once	Twice or more
15-How many times does your child brush a day?	296 (67.12%)	140 (31.75%)	5 (1.13%)

### Associations

#### A-Correlations between oral manifestations and age

None of the correlations were statistically significant (*p* > 0.05); also, the correlation between total OAG and age was not statistically significant (*p* > 0.05), as presented in Table 5.

**Table 5:** Correlations between oral manifestation scores and age

Parameter	Correlation coefficient (95% CI)	*p*-value
Voice	-0.021 (-0.134:0.093)	0.722ns
Swallow	-0.059 (-0.172:0.054)	0.305ns
Lips	0.105 (-0.009:0.215)	0.070ns
Tongue	0.064 (-0.049:0.176)	0.267ns
Saliva	0.050 (-0.064:0.162)	0.393ns
Mucous Membrane	-0.018 (-0.131:0.095)	0.752ns
Gingiva	0.037 (-0.076:0.150)	0.522ns
Teeth	0.099 (-0.015:0.210)	0.088ns
Total OAG score	0.066 (-0.047:0.178)	0.254ns
Mucositis	-0.083 (-0.194:0.031)	0.152ns

^*^Significant (*p* < 0.05) ns, non-significant (*p* > 0.05)

#### B-Associations between oral manifestations and gender

**Fig. 5:**
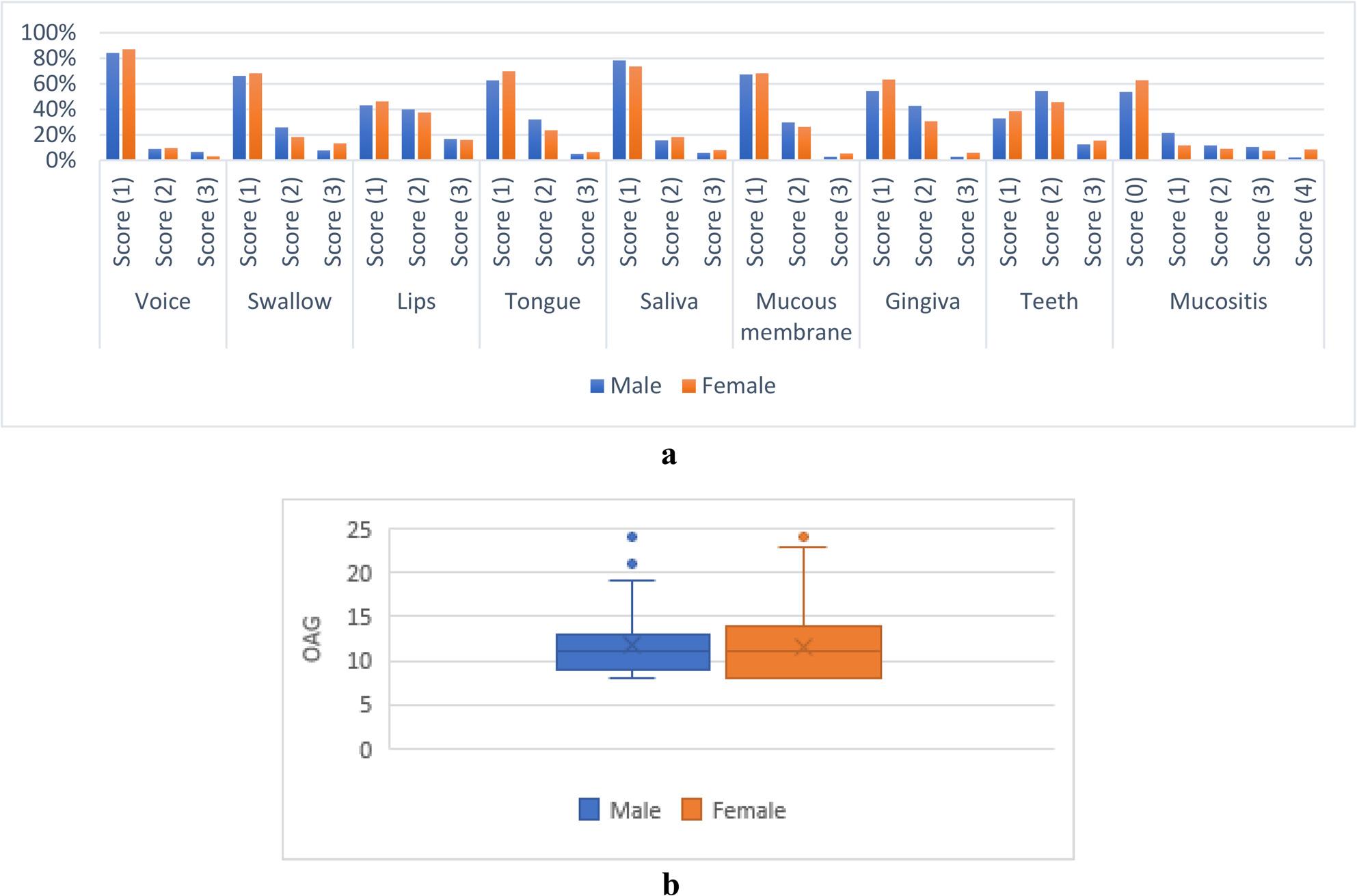
**a** Associations between oral manifestations and gender. **b** OAG score values for different genders

None of the associations were statistically significant (*p* > 0.05). Associations between oral manifestations and gender are presented in Fig. 5.

#### C-Associations between oral manifestations score and type of cancer

**Fig. 6:**
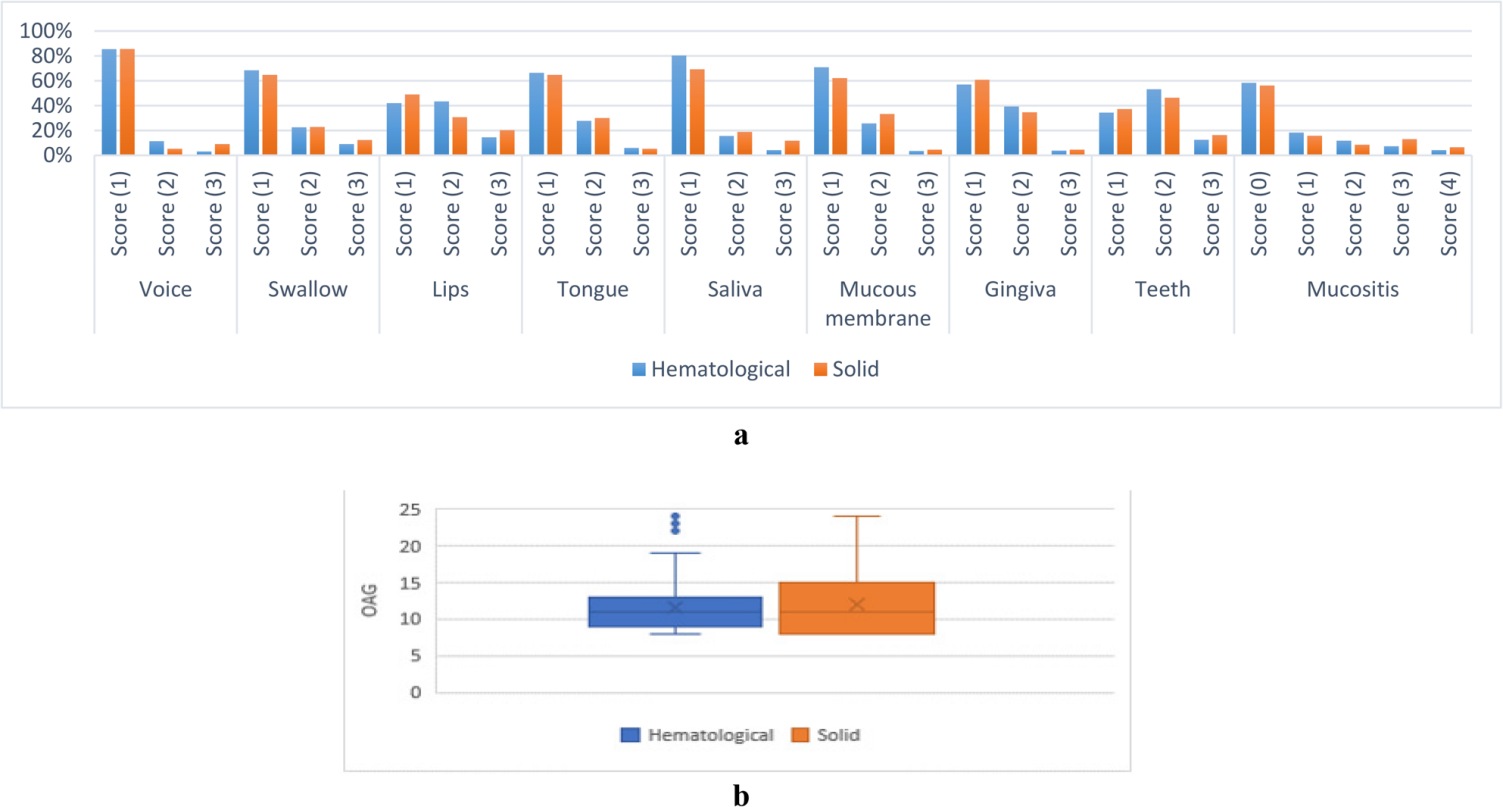
**a** Associations between oral manifestations score and type of cancer. **b** OAG score values for different types of cancer

A significant association was found between the type of cancer and saliva score, with cases of solid tumors exhibiting significantly higher scores than those with hematological lesions (*p* = 0.005). Other correlations were not statistically significant (*p* > 0.05) as presented in Fig. 6.

#### D-Associations between total OAG score and answers to parental dental awareness questionnaire

**Fig. 7:**
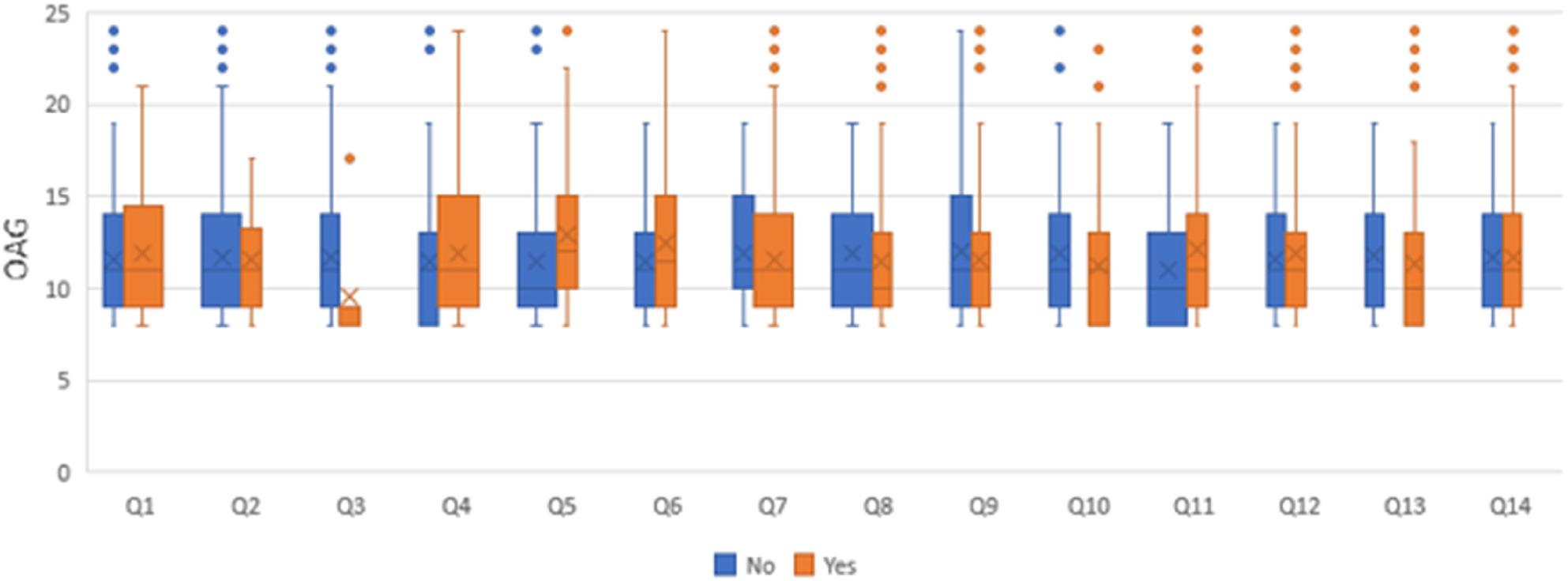
OAG score values for different answers to the parental awareness questionnaire

For question (3), respondents choosing “No” had significantly higher total OHG scores than those who chose “Yes” (*p* = 0.039). For questions (5), (6), and (11), respondents who chose “Yes” had significantly higher scores (*p* < 0.05). For other questions, the associations were not statistically significant (*p* > 0.05) as presented in Fig. 7.

#### E-Associations between oral manifestation scores and teeth brushing per day

**Fig. 8:**
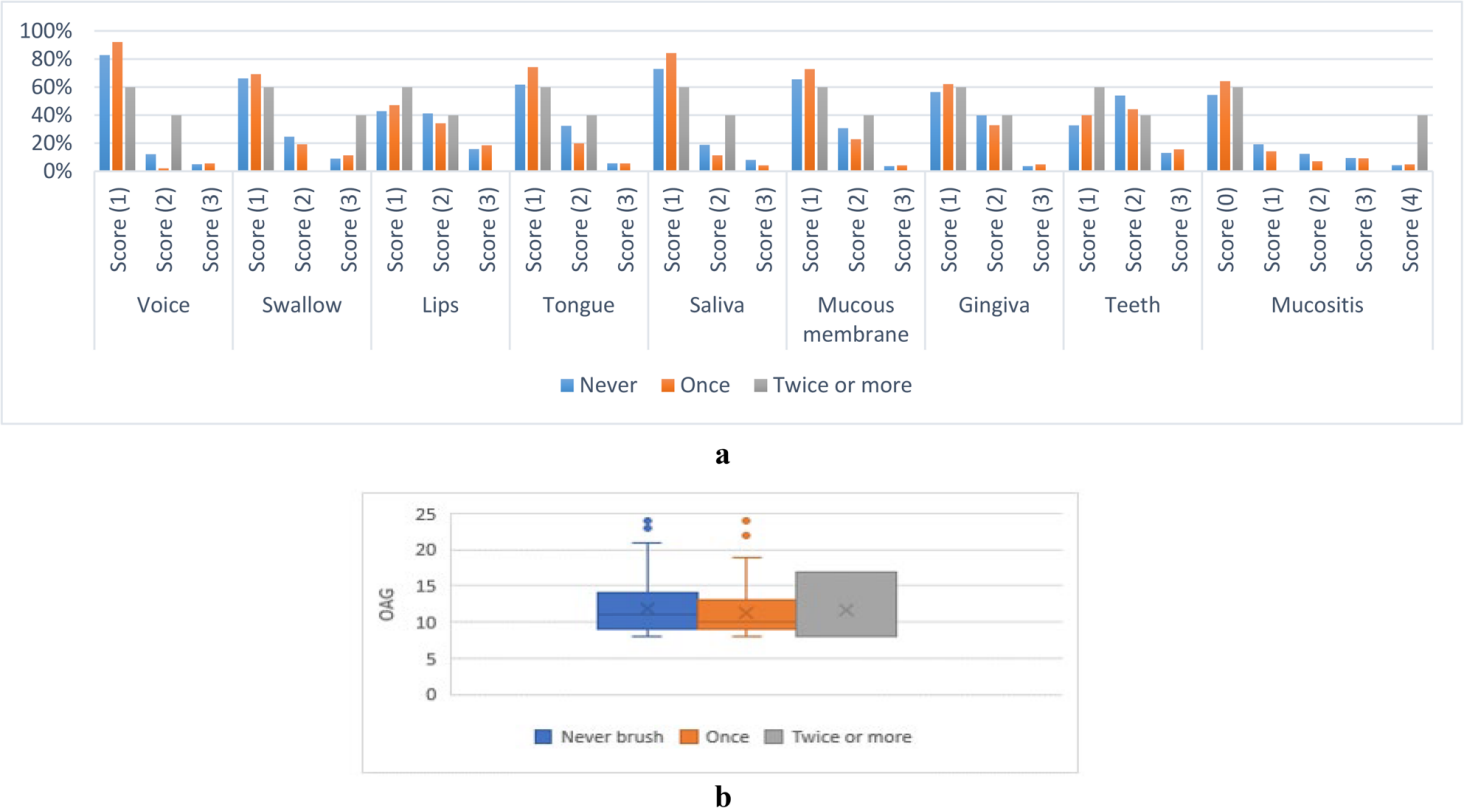
**a **Associations between oral manifestation scores and brushing frequency per day. **b** OAG score values for different brushing frequencies per day

For voice and saliva scores, children who brushed their teeth once had significantly lower scores than those who did not (*p* < 0.05). For other parameters, the associations were not statistically significant (*p* > 0.05) as presented in Fig. 8.

## Discussion

Oral complications are a well-documented side effect of chemotherapy, particularly among pediatric cancer patients. As per earlier investigations, the prevalence of oral complications arising from chemotherapy treatment ranged from 30% to 100%, positioning these children as a high-risk group due to the rapid turnover rate of oral mucosal cells. The patient with cancer faces an assault on oral health from both the disease and the treatment options available [16]. Consequently, early oral examination and management, along with proper hygiene, are essential in reducing morbidity and improving patient outcomes [17].

In the current study, the Oral Assessment Guide (OAG) was employed because of its clinical simplicity and comprehensiveness in evaluating both functional and physical aspects of the oral cavity. While the OAG facilitates quick assessments across eight aspects, it does not provide specific measurements of lesion size or extent. It does not distinguish between various regions of the mucous membranes [18]. To address these limitations, the WHO Mucositis Scale was also incorporated, providing an objective, valid, and reproducible assessment suitable for routine use in clinical practice [19].

This study employed a pre-tested, structured questionnaire, designed and validated by Gupta et al. [15], to assess caregivers’ awareness of oral health. Our study comprised 441 children and their caregivers, predominantly male (57.82%), with a mean age of 7.64 ± 3.46 years. These demographics are consistent with previous studies, such as those by Garvin [20] and Soliman et al. [21], which have noted a male predominance in pediatric cancers. Biological factors, such as hormonal influences and sex-based immunological variations, may partly explain the observed male dominance [22].

The most prevalent hematological malignancy was leukemia (79.51%), with osteosarcoma (30.72%) leading among solid tumors. These results were in line with those of Soliman et al. [21] and Taran et al. [23]. The unique biology of blood cell development in children, the frequency of cell division in bone marrow, and the biological susceptibility during times of fast bone growth throughout puberty, along with the fact that osteosarcoma originates from bone-forming cells that are vulnerable to malignant transformation, are the main reasons why they are considered the most frequent malignancies in children [24, 25].

The most common complication, according to the OAG scores, was tooth debris, followed by involvement of the lips, gingiva, and tongue. The mean OAG score was 11.66, which is consistent with the findings of Chen et al. [26]. Cancer patients frequently have dental debris accumulation as a result of immunological suppression, dry mouth brought on by treatment, mucositis-related oral pain, and dietary variables that compromise oral hygiene and encourage plaque retention. To prevent the development of more serious dental complications, strict dental care and monitoring are necessary throughout cancer treatment [27].

As indicated by our investigation, the prevalence rate of mucositis among pediatric patients undergoing chemotherapy was 42.4%, representing those who experienced varying degrees of mucositis during their treatment. This rate was relatively lower, yet comparable to findings from other studies, which ranged from 51.7% to 75% [28–32]. These differences could be attributed to variations in grading scales, assessment tools, and oral care practices.

Our findings revealed that 67.12%of children never brushed their teeth, which corresponded to a 75.74% incidence of mucositis during chemotherapy. Alkhuwaiter [33] similarly found inadequate oral hygiene practices during chemotherapy, though with slightly better parental involvement. The reason behind this was that the guardians of these children exhibit heightened apprehension towards the prevailing illness of their offspring, often disregarding the significance of oral hygiene. This tendency can be attributed to their limited understanding of the necessity of establishing a dental routine and scheduling regular dental check-ups, only realizing its importance when faced with a sudden dental emergency.

Moreover, this also reflects the reason why the parents of 108 children were not concerned about their children’s dental treatment, and their response to the preventive advice was poor [33]. These outcomes underscore the importance of caregiver education in preventive oral care.

Despite recommendations, 95.01% of caregivers failed to take their child to the dentist before initiating chemotherapy, indicating a lack of awareness; these findings were comparable to those of Gupta et al. and Alkhuwaiter [15, 33]. Bleeding gums (21.77%) were frequently reported, most likely due to chemotherapy-induced thrombocytopenia, exacerbated by poor oral hygiene [34]. These results were similar to those reported by Alkhuwaiter [33], who found gingival bleeding in 19.5% of the cases.

Preventive interventions, such as fluoride application, were underutilized, with 98.41% of patients lacking a history of application. Only 14.51% of affected children were referred for emergency dental care. Pedrosa et al. reported similar limited utilization [35], highlighting systemic gaps in dental referral protocols during cancer care.

Nutritional counseling was provided to 75.51% of caregivers, recognizing its importance in enhancing treatment tolerance, infection susceptibility, and quality of life [36]. However, practices such as mouthwash use (32.88%) and daily oral rinses (20.41%) were infrequent, often delayed until complications emerge. Fear of unintentional ingestion was a frequently expressed concern [33].

Dietary habits revealed that 47.39% of children consumed sugary snacks between meals, a lower percentage than Alkhuwaiter [33], possibly reflecting differences in parental attitudes or socioeconomic factors. Poor dietary control, along with inadequate hygiene, tends to worsen the oral manifestations during treatment [31, 37].

Statistical analysis revealed no significant correlation between oral manifestations and age or gender, aligning with previous research findings [37, 38, 39]. However, Harada et al. [40] and Martins et al. [41] suggested that younger children are more vulnerable to mucositis due to higher rates of cell turnover, though they also heal faster.

Specific OAG scores and caregiver questionnaire responses demonstrated significant correlations, particularly in relation to brushing frequency and awareness of the condition. This reinforces the conception that caregiver knowledge and attitudes strongly influence pediatric oral health during chemotherapy [42].

This study contributes to the international literature by integrating clinical assessment tools (OAG and WHO Mucositis Scale) with a caregiver awareness questionnaire to thoroughly evaluate oral health in pediatric oncology. It highlights critical gaps in parental awareness, preventive care, and dental referral procedures, areas that remain insufficiently researched in low- and middle-income countries. By doing so, it contributes to the formulation of more effective, multidisciplinary interventions to mitigate oral complications and improve the quality of life for children undergoing chemotherapy.

### Limitations

1. Other variables that might have an impact on the onset of the oral manifestations and the oral health outcomes were not considered for each patient (The type, dosage, and schedule of administration of chemotherapy, the renal function, and hematological parameters of the patient).
2. The mucosal status at the time of examination might not reflect the full impact of chemotherapy, since the presentation of OM varies throughout the course of chemotherapy.
3. This study was conducted in a single hospital, not a multicenter initiative.
4. Lack of socio-demographic data of the parents, to study the correlation between socio-demographic data and the level of knowledge.

## Conclusion

Antineoplastic therapy may negatively impact a cancer patient’s oral health, potentially having a profound effect on the patient’s overall well-being. The most common oral manifestation seen was teeth debris, and the least common was voice alteration. Many patients had oral complaints and received dietary counseling from the pediatrician or dietitian; parents were also interested in dental care for their children. On the other hand, parents lacked sufficient awareness regarding dental care. Therefore, it is crucial to schedule an appointment with a pediatric dentist for a thorough dental evaluation and treatment before initiating chemotherapy. Through interprofessional collaborative care, patient comfort and optimal outcomes are ensured during and after cancer therapy. Future objectives involve crucial interventions to educate, guide, and motivate patients, their family members, and nursing staff to maintain a satisfactory level of oral hygiene, especially during hospitalization.

## Data Availability

The datasets used are available from the corresponding author on reasonable request.

## Abbreviations

OAG: Oral Assessment Guide
WHO: World Health Organization
DNA: Deoxyribonucleic Acid
OM: Oral Mucositis
ALL: Acute Lymphoblastic Leukemia

## References

[1] Seyfried TN, Shelton LM. Cancer as a metabolic disease. Nutr Metab (Lond). 2010;7. 10.1186/1743-7075-7-7. 10.1186/1743-7075-7-7 PMC2845135 20181022

[2] Ward ZJ, Yeh JM, Bhakta N, Frazier AL, Atun R. Estimating the total incidence of global childhood cancer: a simulation-based analysis. Lancet Oncol. 2019;20:483–93. 10.1016/S1470-2045(18)30909-4. 30824204 10.1016/S1470-2045(18)30909-4

[3] El Malla H, Steineck G, Ylitalo Helm N, Wilderäng U, El Sayed Elborai Y, Elshami M, et al. Cancer disclosure account from a pediatric oncology ward in Egypt. Psychooncology. 2017;26:679–85. 10.1002/PON.4207. 27362448 10.1002/pon.4207

[4] Mack JW, Fasciano KM, Block SD. Communication about prognosis with adolescent and young adult patients with cancer: Information needs, prognostic awareness, and outcomes of disclosure. J Clin Oncol. 2018;36:1861–7. 10.1200/JCO.2018.78.2128. 29683788 10.1200/JCO.2018.78.2128

[5] Fadhil I, Soliman R, Jaffar S, Al Madhi S, Saab R, Belgaumi A, et al. Estimated incidence, prevalence, mortality, and registration of childhood cancer (ages 0–14 years) in the WHO Eastern Mediterranean region: an analysis of GLOBOCAN 2020 data. Lancet Child Adolesc Health. 2022;6:466–73. 10.1016/S2352-4642(22)00122-5. 35605628 10.1016/S2352-4642(22)00122-5

[6] Van den Boogaard WMC, Komninos DSJ, Vermeij WP. Chemotherapy Side-Effects: Not All DNA Damage Is Equal. Cancers (Basel). 2022;14. 10.3390/CANCERS14030627. 10.3390/cancers14030627 PMC8833520 35158895

[7] Triarico S, Agresti P, Rinninella E, Mele MC, Romano A, Attinà G, et al. Oral Microbiota during Childhood and Its Role in Chemotherapy-Induced Oral Mucositis in Children with Cancer. Pathogens. 2022;11:448. 10.3390/PATHOGENS11040448. 35456122 10.3390/pathogens11040448 PMC9025665

[8] Fiwek P, Emerich K, Irga-Jaworska N, Pomiecko D. Photobiomodulation Treatment in Chemotherapy-Induced Oral Mucositis in Young Haematological Patients—A Pilot Study. Med (Lithuania). 2022;58. 10.3390/MEDICINA58081023. 10.3390/medicina58081023 PMC9412323 36013491

[9] Sampaio MEA, Ribeiro ILA, Santiago BM, Valença AMG. Perception of Pediatric Oncological Patients and Their Parents/Guardians about a Hospital Oral Health Program: A Qualitative Study. Asian Pac J Cancer Prev. 2022;23:451–7. 10.31557/APJCP.2022.23.2.451. 35225456 10.31557/APJCP.2022.23.2.451 PMC9272641

[10] Śledzińska A, Śledzińska P, Bebyn M, Komisarek O. Chemotherapy-Induced Oral Complications and Prophylaxis Strategies. Cancer Invest. 2023;41:432–55. 10.1080/07357907.2023.2188558. 36892292 10.1080/07357907.2023.2188558

[11] Hendrawati S, Nurhidayah I, Mediani HS, Mardhiyah A. The Incidence of Mucositis in Children with Chemotherapy Treatment. J Nurs Care. 2019. 10.24198/JNC.V2I1.20129. 2.

[12] Kusiak A, Alicjajereczek-Fossa B, Cichońska D, Alterio D. Oncological-therapy related oral mucositis as an interdisciplinary problem—literature review. Int J Environ Res Public Health. 2020;17. 10.3390/IJERPH17072464. 10.3390/ijerph17072464 PMC7177874 32260309

[13] Eilers J, Berger AM, Petersen MC. Development, testing, and application of the oral assessment guide. Oncol Nurs Forum.1988;15(3):325–30. 3287344

[14] Jaroneski LA. The importance of assessment rating scales for chemotherapy-induced oral mucositis. Oncol Nurs Forum. 2006;33:1085–90. 10.1188/06.ONF.1085-1093. 17149392 10.1188/06.ONF.1085-1093

[15] Gupta A, Marwaha M, Bansal K, Sachdeva A, Gupta A. Dental awareness among parents and oral health of paediatric cancer patients receiving chemotherapy. J Clin Diagn Res. 2016;10:ZC92–5. 10.7860/JCDR/2016/17412.7819. 27437369 10.7860/JCDR/2016/17412.7819 PMC4948545

[16] Mathur VP, Dhillon JK, Kalra G. Oral Health in Children with Leukemia. Indian J Palliat Care. 2012;18:12. 10.4103/0973-1075.97343. 22837605 10.4103/0973-1075.97343 PMC3401728

[17] Azher U, Shiggaon N. Oral health status of children with acute lymphoblastic leukemia undergoing chemotherapy. Indian J Dent Res. 2013;24:523. 10.4103/0970-9290.118371. 24047855 10.4103/0970-9290.118371

[18] Eilers J, Million R. Prevention and Management of Oral Mucositis in Patients With Cancer. Semin Oncol Nurs. 2007;23:201–12. 10.1016/j.soncn.2007.05.005. 17693347 10.1016/j.soncn.2007.05.005

[19] Lalla RV, Sonis ST, Peterson DE. Management of Oral Mucositis in Patients Who Have Cancer. Dent Clin North Am. 2008;52:61–77. 10.1016/J.CDEN.2007.10.002. 18154865 10.1016/j.cden.2007.10.002 PMC2266835

[20] Garvin JH. Gender-Specific Aspects of Pediatric Hematology and Oncology. Principles Gender-Specific Med. 2010;51–61. 10.1016/B978-0-12-374271-1.00004-6.

[21] Soliman RM, Elhaddad A, Oke J, Eweida W, Sidhom I, Ahmed S, et al. Temporal trends in childhood cancer survival in Egypt, 2007 to 2017: A large retrospective study of 14,808 children with cancer from the Children’s Cancer Hospital, Egypt. Int J Cancer. 2021;148:156274. 10.1002/IJC.33321. 10.1002/ijc.33321 32997796

[22] Lopes-Ramos CM, Quackenbush J, DeMeo DL. Genome-Wide Sex and Gender Differences in Cancer. Front Oncol. 2020;10:597788. 10.3389/FONC.2020.597788/XML. 33330090 10.3389/fonc.2020.597788 PMC7719817

[23] Taran SJ, Taran R, Malipatil NB. Pediatric osteosarcoma: An updated review. Indian J Med Pediatr Oncol. 2017;38:33–43. 10.4103/0971-5851.203513. 10.4103/0971-5851.203513 PMC5398104 28469335

[24] Ivan A, Cojocaru E, Sirbu PD, Al Namat DR, Tîrnovanu ȘD, Butnariu LI, Bernic J, Bernic V, Țarcă E. Clinical and Pathological Profile of Children and Adolescents with Osteosarcoma. Diagnostics. 2025;15:266. 10.3390/diagnostics15030266. 39941196 10.3390/diagnostics15030266 PMC11817002

[25] Wiemels J. Perspectives on the causes of childhood leukemia. Chem Biol Interact. 2012;196(3):59–67. 10.1016/j.cbi.2012.01.007. Epub 2012 Feb 2. PMID: 22326931; PMCID: PMC3839796. 22326931 10.1016/j.cbi.2012.01.007 PMC3839796

[26] Chen CF, Wang RH, Cheng SN, Chang YC. Assessment of chemotherapy-induced oral complications in children with cancer. J Pediatr Oncol Nurs. 2004;21:33–9. 10.1177/1043454203259947. 15058405 10.1177/1043454203259947

[27] Benito-Ramal E, Camacho-Mourelo A, González-Navarro B, López JL, Jané-Salas E. Prevalence and risk factors of chronic oral complications in head and neck cancer therapies: A retrospective study. Med Oral Patol Oral Cir Bucal. 2024;29(6):e850–6. 26823. PMID: 39396141; PMCID: PMC11584959. 39396141 10.4317/medoral.26823 PMC11584959

[28] Mazhari F, Shirazi AS, Shabzendehdar M. Management of oral mucositis in pediatric patients receiving cancer therapy: A systematic review and meta-analysis. Pediatr Blood Cancer. 2019;66. 10.1002/PBC.27403. 10.1002/pbc.27403 30421549

[29] Guggenheimer J, Verbin RS, Appel BN, Schmutz J. Clinicopathologic effects of cancer chemotherapeutic agents on human buccal mucosa. Oral Surgery, Oral Medicine. Oral Pathol. 1977;44:58–63. 10.1016/0030-4220(77)90244-4. 10.1016/0030-4220(77)90244-4 267885

[30] Janković L, Jelić S, Filipović-Lješković I, Ristović Z. Salivary immunoglobulins in cancer patients with chemotherapy-related oral mucosa damage. Eur J Cancer B Oral Oncol. 1995;31:160–5. 10.1016/0964-1955(95)00011-6. 10.1016/0964-1955(95)00011-6 7549754

[31] Gandhi K, Datta G, Ahuja S, Saxena T, Datta AG. Prevalence of Oral Complications occurring in a Population of Pediatric Cancer Patients receiving Chemotherapy. Int J Clin Pediatr Dent. 2017;10:166–71. 10.5005/ID-IOURNALS-10005-1428. 28890617 10.5005/iD-iournals-10005-1428 PMC5571386

[32] WAHLIN YB. Oral mucosal lesions in patients with acute leukemia and related disorders during cytotoxic therapy. Eur J Oral Sci. 1988;96:128–36. 10.1111/J.1600-0722.1988.TB01419.X. 10.1111/j.1600-0722.1988.tb01419.x 3258443

[33] Alkhuwaiter S. Parents’ Awareness and Oral Health Care Measures of Pediatric Patients Receiving Chemotherapy. J Pediatr Dentistry. 2021. 10.14744/JPD.2021.04_38.

[34] Francisconi CF, Caldas RJ, Martins LJO, Rubira CMF, Santos PS da S. Leukemic oral manifestations and their management. Asian Pac J Cancer Prev. 2016;17:911–5. 10.7314/APJCP.2016.17.3.911. 27039811 10.7314/apjcp.2016.17.3.911

[35] PEDROSA BRV, MARTINS WLL, ALMEIDA HCR de. KOZMHINSKY VM da R, SABINO M de FP de A, OLIVEIRA KMM de. Parents’ knowledge about the oral health care of oncological children. RGO - Revista Gaúcha de Odontologia. 2019;67:e2019008. 10.1590/1981-86372019000083605.

[36] Barr RD, Stevens MCG. The influence of nutrition on clinical outcomes in children with cancer. Pediatr Blood Cancer. 2020;67. 10.1002/PBC.28117. 10.1002/pbc.28117 32134218

[37] Subramaniam P, Girish Babu KL, Nagarathna J. Oral manifestations in acute lymphoblastic leukemic children under chemotherapy. J Clin Pediatr Dentistry. 2008;32:319–24. 10.17796/JCPD.32.4.0P1462T621W20477. 10.17796/jcpd.32.4.0p1462t621w20477 18767465

[38] Ponce-Torres E, Ruiz-Rodríguez MDS, Alejo-González F, Hernández-Sierra JF, De Pozos-Guillén AD. Oral manifestations in pediatric patients receiving chemotherapy for acute lymphoblastic leukemia. J Clin Pediatr Dentistry. 2010;34:275–9. 10.17796/JCPD.34.3.Y060151580H301T7. 10.17796/jcpd.34.3.y060151580h301t7 20578668

[39] Ramírez-Amador V, Esquivel-Pedraza L, Mohar A, Reynoso-Gómez E, Volkow-Fernández P, Guarner J, et al. Chemotherapy-associated oral mucosal lesions in patients with leukaemia or lymphoma. Eur J Cancer B Oral Oncol. 1996;32:322–7. 10.1016/0964-1955(96)00020-6. 10.1016/0964-1955(96)00020-6 8944835

[40] Harada K, Ferdous T, Horinaga D, Uchida K, Mano T, Mishima K, et al. Efficacy of elemental diet on prevention for chemoradiotherapy-induced oral mucositis in patients with oral squamous cell carcinoma. Support Care Cancer. 2016;24:953–9. 10.1007/S00520-015-2866-7. 26248650 10.1007/s00520-015-2866-7

[41] Martins A, de CM, Caçador NP, Gaeti WP. Complicações bucais da quimioterapia antineoplásica. Acta Scientiarum Health Sci. 2008;24:663–70. 10.4025/actascihealthsci.v24i0.2481.

[42] Nessa J, Jahan I, Asha MS, Anika SA, Zakir Hossain Shikder AH. Prevention of Oral Manifestations in Acute Lymphoblastic Leukemic Children (ALL) by Meticulous Mouth Preparation including Fluoride Application. J Community Med Public Health Rep. 2022. 10.38207/JCMPHR/2022/SEP03070298.

